# Inactivation of CACNA1H induces cell apoptosis by initiating endoplasmic reticulum stress in glioma

**DOI:** 10.1515/tnsci-2022-0285

**Published:** 2023-05-26

**Authors:** Sheng Liu, Ying Ba, Chenglong Li, Guangming Xu

**Affiliations:** Department of Neurosurgery, Binzhou Medical University Hospital, Binzhou, 256603, China; Department of Gastroenterology, Binzhou Medical University Hospital, Binzhou, 256603, China; Department of Neurosurgery, Shandong Provincial Hospital, Shandong University, No. 324, Jingwuweiqi Road, Jinan, 250021, China

**Keywords:** T-type Ca^2+^ channel, glioma, endoplasmic reticulum stress, apoptosis, Cav3.2

## Abstract

**Background:**

Ca^2+^ channels are abnormally expressed in various tumor cells and are involved in the progression of human glioma. Here, we explored the role of a calcium channel, voltage-dependent, T-type, alpha 1H subunit (CACNA1H), which encodes T-type Ca^2+^ channel Cav3.2 in glioma cells.

**Methods:**

Cell viability and apoptosis were detected using cell-counting kit-8 and flow cytometry, respectively. The expression of target protein was determined using western blot analysis.

**Results:**

Cell viability of U251 cells was inhibited significantly after the knockdown of CACNA1H. The apoptosis of U251 cells was enhanced significantly after the knockdown of CACNA1H. Importantly, knockdown of CACNA1H decreased the levels of p-PERK, GRP78, CHOP, and ATF6, indicating that CACNA1H knockdown activated endoplasmic reticulum stress (ERS) in U251 cells. In addition, T-type Ca^2+^ channel inhibitor NNC55-0396 also induced apoptosis through the activation of ERS in U251 cells. ERS inhibitor UR906 could block CACNA1H inhibitor ABT-639-induced apoptosis.

**Conclusion:**

Suppression of CACNA1H activated the ERS and thus induced apoptosis in glioma cells. T-type Ca^2+^ channel inhibitors ABT-639 and NNC55-0396 also induced apoptosis through ERS in glioma cells. Our data highlighted the effect of CACNA1H as an oncogenic gene in human glioma.

## 1 Introduction

Gliomas are the most common central nervous system malignancies, which are characterized by low surgical resection rate, high recurrence, and poor sensitivity to chemoradiotherapy [1]. Intracellular Ca^2+^ is an important signal regulating cell proliferation, differentiation, and apoptosis [2,3]. Ca^2+^ channels are abnormally expressed in various tumor cells and are involved in the progression of human glioma [4,5]. T-type Ca^2+^ channels are low-voltage-gated channels that are widely expressed in normal tissues, including brain, kidney, heart, and smooth muscle [6,7]. Studies have shown that T-type Ca^2+^ channels are highly expressed in various types of gliomas, and their increased expression can promote tumor cell proliferation [6]. Valerie et al. found that treatment with the T-type selective antagonist mibefradil or knockdown of T-type Ca^2+^ channel reduced cell viability and induced apoptosis in U251, T98G, and U87 cells [8]. At present, the specific mechanism by which T-type Ca^2+^ channel regulates glioma progression remains unclear.

T-type Ca^2+^ channels include three subtypes, Cav3.1, Cav3.2, and Cav3.3, encoded by CACNA1G, CACNA1H, and CACNA1I, respectively [9]. Studies have found that CACNA1H mutations exist in childhood absence epilepsy [10], generalized epilepsy with febrile seizures plus [11], and autism spectrum disorders [12], suggesting that CACNA1H plays an important role in neurological lesions. NNC-55 (T-channel inhibitor) or knockdown of CACNA1H can activate endoplasmic reticulum stress (ERS), thus increasing autophagy inhibition and apoptotic signals in CACNA1H−/− mice [13]. Tumor cells are maintained in a hypoxic, glucose-deficient, low pH microenvironment due to their rapid growth, which induces ERS and unfolded protein responses (UPRs) [14]. ERS participates in the process of tumorigenesis, development, and drug resistance by inducing autophagy, invasion, and apoptosis of tumor cells [15]. However, there is no report on whether CACNA1H participates in tumor progression through ERS. Here, we investigated this hypothesis in a glioma cell model *in vitro*.

## 2 Materials and methods

### 2.1 Cell culture and treatment

The U251 human glioma cells were cultured in Dulbecco’s Modified Eagle Medium supplemented with 10% fetal bovine serum (Thermo Fisher Scientific, Shanghai, China). CACNA1H-specific small interfering RNA (siRNA) was synthesized in GeneChem Co., Ltd (Shanghai, China) and transfected into the U251 cells using lipo2000 (Invitrogen, Carlsbad, CA, USA) according to the manufacturer’s instruction. NNC 55-0396 hydrate (NNC-55), ABT-639, and UR906 were purchased from MedChemExpress (Monmouth Junction, NJ, USA). Cells were seeded into the six-well plate and treated with NNC-55 (10 μM), ABT-639 (10 μM), or UR906 (30 μM) for 12 h.

### 2.2 Detection of cell viability

Cell-counting kit-8 (CCK8) (MedChemExpress, Monmouth Junction, NJ, USA) assay was performed to determine cell viability. The U251 cells (1,000 cells/well) were transferred into a 96-well plate. For viability detection, cells were incubated with 10 μl of CCK8 for 1.5 h at 37°C. Optical density value at 450 nm was measured using a microplate reader.

### 2.3 Western blot analysis

Total protein was extracted from U251 cells using RIPA buffer and separated using sodium dodecyl sulfate-polyacrylamide gel electrophoresis. Then, protein samples were transferred to a polyvinylidene difluoride membrane. After blocking with 5% non-fat milk, the membrane was incubated with primary antibodies at 4℃ overnight followed by secondary antibodies at room temperature for 1 h. The primary antibodies Bcl-2 (26593-1-AP), GRP78 (11587-1-AP), CHOP (15204-1-AP), and ATF6 (24169-1-AP) were purchased from PTG (Chicago, IL, USA). The primary antibodies pro-Caspase12 (35965), Cleaved-Caspase12 (35965), p-PERK (3179), and PERK (3192) were purchased from Cell Signaling Technology (Beverly, MA, USA). The specific bands were visualized using an enhanced chemiluminescence kit (Solarbio Science & Technology Co. Ltd., Beijing, China). ImageJ software was used for the quantification of target protein.

### 2.4 Apoptosis detection

Flow cytometry was performed to detect cell apoptosis using the AnnexinV-FITC/PI kit (Beijing 4A Biotech Co., Ltd, Beijing, China) according to the operating instruction. Cell signal was collected using flow cytometry (BD, Franklin Lakes, NJ, USA). Apoptotic cells were analyzed using FlowJo software (BD Biosciences).

### 2.5 Data analysis

Data were analyzed using GraphPad Prism 9 and presented as mean ± SD. Student’s *t* test and one-way analysis of variance with post-hoc Bonferroni were used for data analysis. *P* < 0.05 was considered statistically significant.

## 3 Results

### 3.1 Knockdown of CACNA1H inhibits proliferation and induces apoptosis in glioma cells

**Figure 1:**
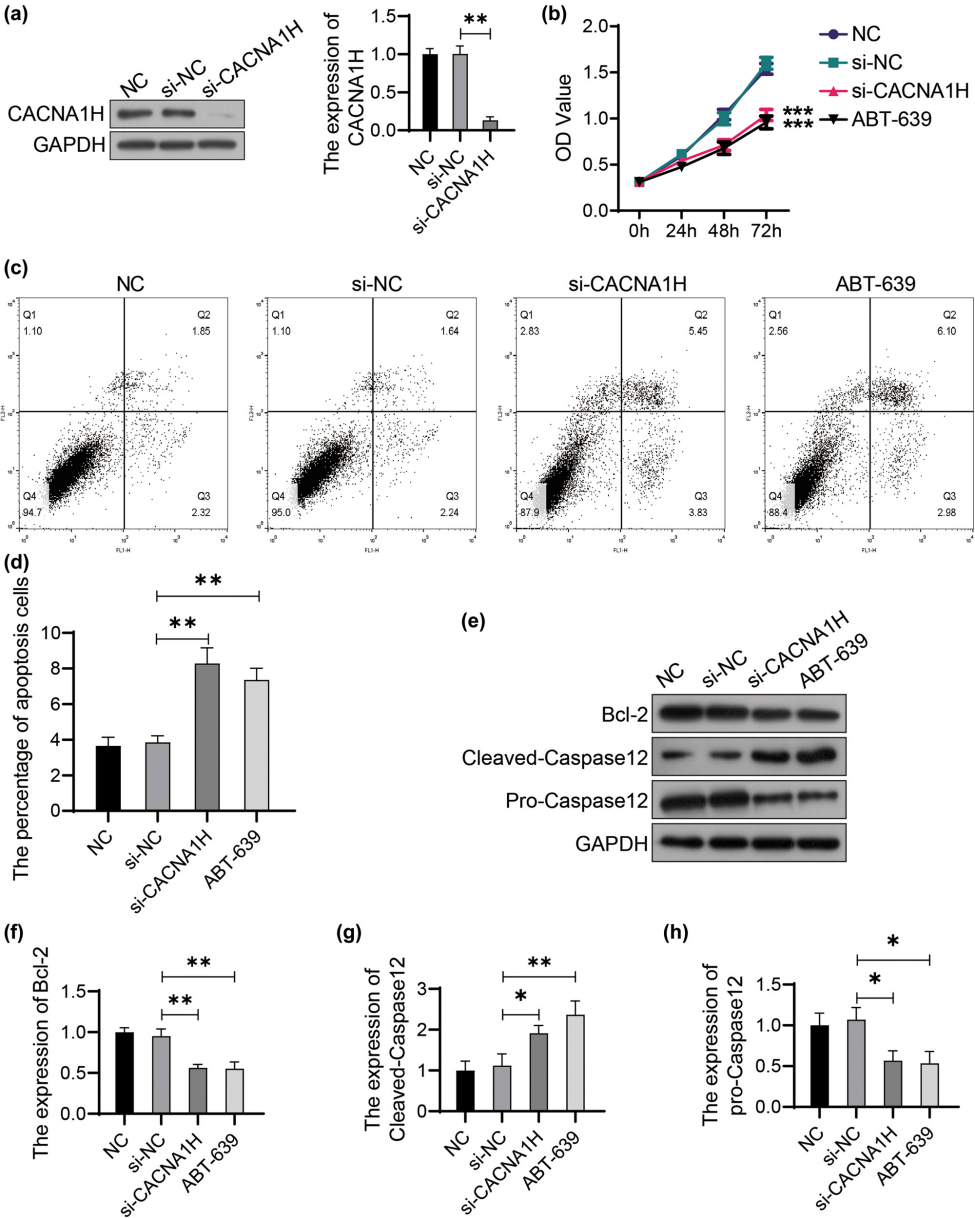
Knockdown of CACNA1H inhibits proliferation and induces apoptosis in glioma cells. (a) The efficiency of siRNA knockdown was detected using western blot. The U251 cells transfected with nonspecific siRNA were used as the negative control (NC). (b) CACNA1H was knocked down by specific siRNA or ABT-639 (10 μM) in U251 cells. Cell viability was determined using CCK8 assay. (c) Flow cytometry was performed to detect cell apoptosis. (d) Apoptotic cells were analyzed using FlowJo software. (e) The expression of target protein was detected using western bolt. The relative expression of Bcl-2 (f), pro-Caspase12 (g), and Cleaved-Caspase12 (h) was analyzed using ImageJ software. *P < 0.05; **P < 0.01; ***P < 0.001.

To explore the specific role of CACNA1H in glioma cells, we inactivated CACNA1H using siRNA technology (Figure 1a) or CACNA1H inhibitor (ABT-639). ABT-639 is a Cav3.2-specific inhibitor which is less active at other Ca^2+^ channels (Cav3.1 and Cav3.3). The viability decreased significantly 48 h after the knockdown of CACNA1H. The effect of ABT-639 on cell proliferation was consistent with that of CACNA1H siRNA (Figure 1b). On the contrary, the percentage of apoptosis increased significantly after CACNA1H knockdown or ABT-639 treatment (Figure 1c and d). Then we detected apoptosis-related proteins using western blot (Figure 1e). Our results proved that the expression of Bcl-2 (Figure 1f) and pro-Caspase12 (Figure 1g) decreased significantly, while the expression of Cleaved-Caspase12 (Figure 1h) increased significantly in CACNA1H-inactivated U251 cells. Decreased proliferation and increased apoptosis confirmed the inhibitory effect of CACNA1H inactivation on glioma cell growth.

### 3.2 Knockdown of CACNA1H activates ERS in U251 cells

**Figure 2:**
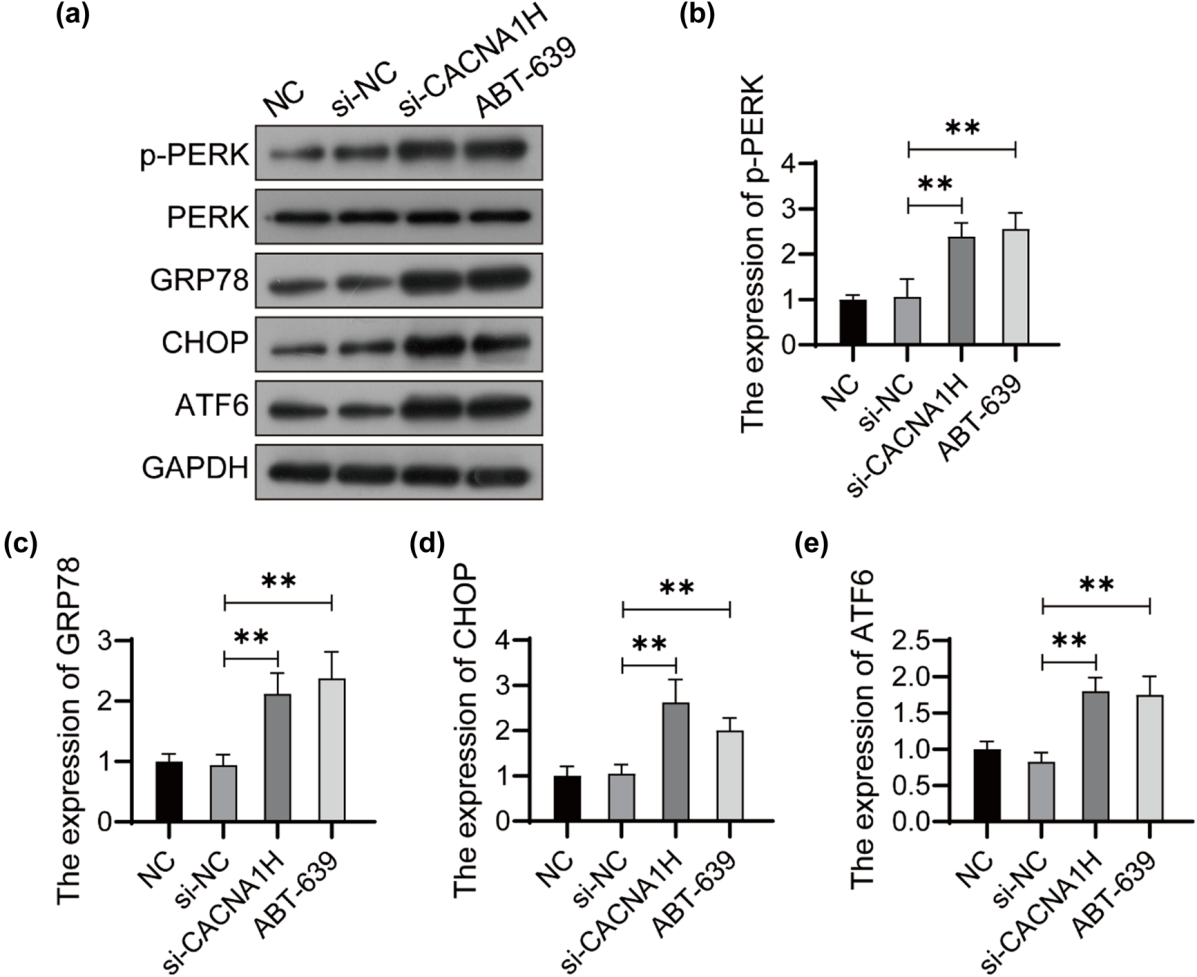
Knockdown of CACNA1H activates ERS in U251 cells. (a) The expression of ERS-related proteins was detected using western bolt. The relative expression of p-PERK (b), GRP78 (c), CHOP (d), and ATF6 (e) was analyzed using ImageJ software. **P < 0.01.

Since knockdown of CACNA1H induced tumor cell apoptosis, we further investigated whether CACNA1H was involved in apoptosis regulation through ERS pathway. As shown in Figure 2, the expression levels of ERS-related proteins were detected using western bolt. The phosphorylation level of PERK increased significantly in CACNA1H knockdown cells compared with that of control cells. The GRP78, CHOP, and ATF6 proteins were upregulated in the CACNA1H knockdown cells. The expression levels of ERS markers p-PERK, GRP78, CHOP, and ATF6 were also inhibited in ABT-639-treated U251 cells (Figure 2).

### 3.3 NNC55-0396 induces apoptosis through the activation of ERS in U251 cells

**Figure 3:**
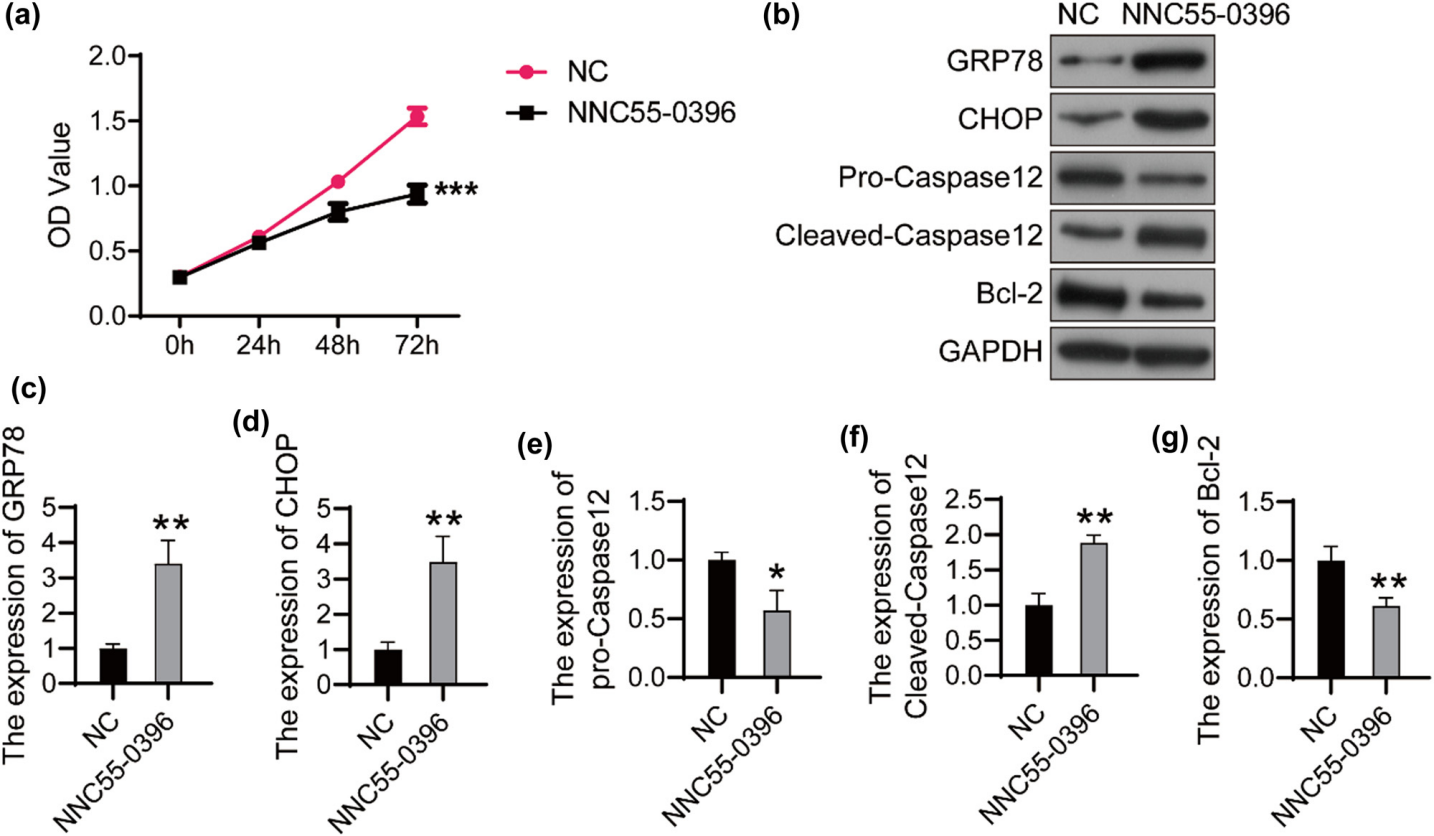
NNC55-0396 activates ERS and induces apoptosis in U251 cells (a) The U251 cells were treated with 10 μM of NNC-55, and cell viability was detected using CCK8 every 24 h. (b) The expression of target proteins was detected using western bolt. The relative expression of GRP78 (c), CHOP (d), pro-Caspase12 (e), Cleaved-Caspase12 (f) and Bcl-2 (g) was analyzed using ImageJ software. *P < 0.05; **P < 0.01; ***P < 0.001.

NNC55-0396 is a potent T-type Ca^2+^ channel inhibitor, which has been found to induce apoptosis of various tumor cells in recent years. Here, we investigated its effect on U251 cells. As shown in Figure 3a, the proliferation of cells treated with NNC55-0396 decreased significantly compared with the control group.

Importantly, the expression of ERS makers GRP78 and CHOP reduced significantly after the treatment with NNC55-0396 (Figure 3b–d). Bcl-2 and pro-Caspase12 were downregulated and Cleaved-Caspase12 was upregulated in NNC55-0396-treated U251 cells (Figure 3e–g). These results indicated that NNC55-0396 induces apoptosis through the activation of ERS in glioma cells.

### 3.4 UR906 blocked the apoptosis induced by ABT-639

**Figure 4:**
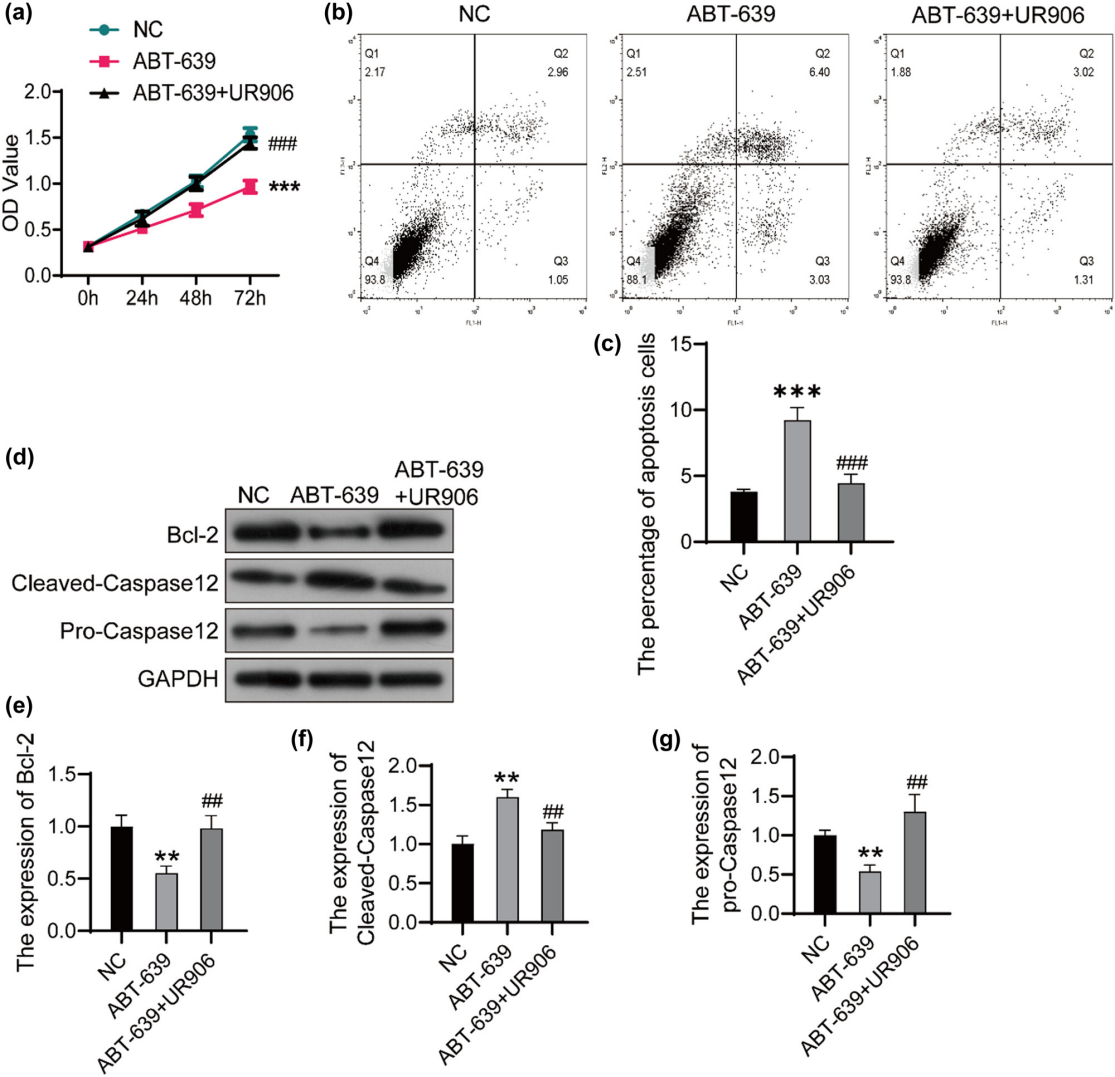
ERS inhibitor UR906 blocked the apoptosis induced by ABT-639. (a) The U251 cells were treated with both ABT-639 (10 μM) and UR906 (30 μM) or only ABT-639 (10 μM) with PBS as the control (NC). Cell viability was determined by CCK8 assay. (b) Flow cytometry was performed to detect cell apoptosis. (c) Apoptotic cells were analyzed using FlowJo software. (d) The expression of target protein was detected using western bolt. The relative expression of Bcl-2 (e), Cleaved-Caspase12 (f), and pro-Caspase12 (g) was analyzed using ImageJ software. **P < 0.01 vs NC group; ***P < 0.001 vs NC group; ^##^ *P* < 0.01 vs ABT-639 group; ^###^ *P* < 0.001 vs ABT-639 group.

Next, we investigated the effect of ERS inhibitor UR906 in U251 cells treated with ABT-639. As shown in Figure 4a, cell proliferation increased significantly after the treatment of UR906 in ABT-639-pretreated U251 cells. As predicted, the percentage of apoptosis decreased significantly after the treatment of UR906 in ABT-639-pretreated U251 cells (Figure 4b and c). UR906 also decreased the expression of Bcl-2 and pro-Caspase12, and reduced the expression of Bcl-2 in ABT-639-pretreated U251 cells (Figure 4d–g).

### 3.5 UR906 blocked the activation of ERS induced by ABT-639

**Figure 5:**
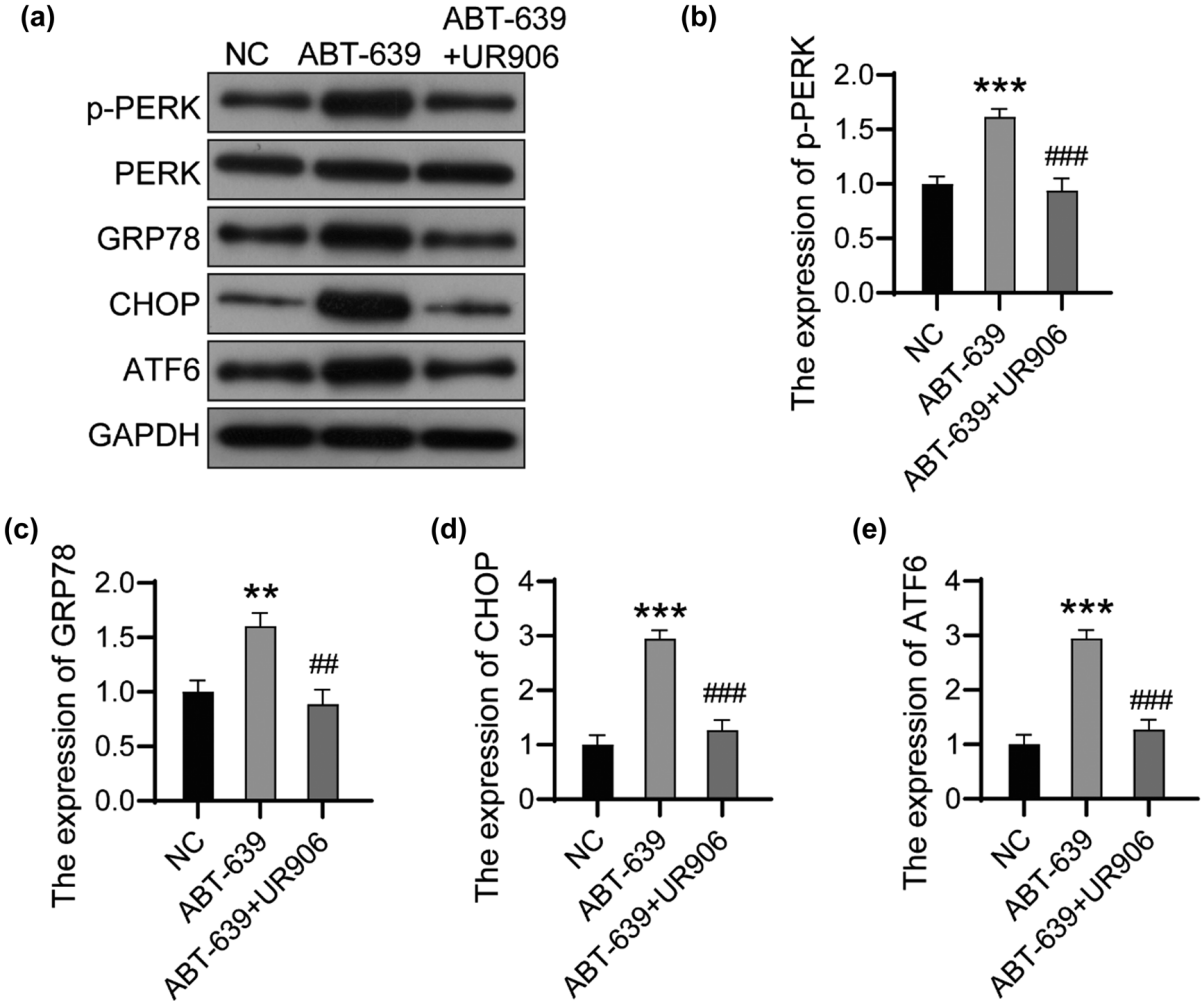
UR906 blocked the activation of ERS induced by ABT-639. (a) The expression of ERS-related proteins was detected using western bolt. The relative expression of p-PERK (b), GRP78 (c), CHOP (d), and ATF6 (e) was analyzed using ImageJ software. **P < 0.01 vs NC group; ***P < 0.001 vs NC group; ^##^ *P* < 0.01 vs ABT-639 group; ^###^ *P* < 0.001 vs ABT-639 group.

Finally, we detected the expression of ERS markers in U251 cells. The phosphorylation level of PERK decreased significantly after the treatment of UR906 in ABT-639-pretreated U251 cells. The GRP78, CHOP, and ATF6 proteins were downregulated after the treatment of UR906 in ABT-639-pretreated U251 cells (Figure 5). These data proved that blocking ERS attenuated CACNA1H knockdown-induced apoptosis in glioma cells.

## 4 Discussion

CACNA1H encodes a low-voltage-activated calcium channel, which is expressed in thalamocortical circuits and is involved in the formation of neuronal burst firing and rhythm generation, as well as sleep and the development of epilepsy [16,17]. In recent years, the role of CACNA1H in tumors has been gradually discovered [18–21]. The genetic defect and mutation of CACNA1H involves in the progression of benign aldosterone-producing tumors [18,22]. As the cellular antenna for breast cancer-specific frequencies, CACNA1H mediates the targeted inhibition of breast cancer brain metastasis [23]. In human glioma, endostatin inhibited proliferation and migration by inhibiting the channel currents of Ca(V) 3.1 and Ca(V) 3.2 [24]. Here, we proved that the knockdown of CACNA1H inhibited proliferation and induced apoptosis in U251 cells. CACNA1H inhibitor ABT639 also suppressed proliferation and increased the percentage of apoptotic cells. Subsequently, we detected the expression of Bcl-2 and Caspase12 and found that the knockdown of CACNA1H could activate Caspase12 and inhibit Bcl-2 expression.

Bcl-2 inhibits cell death caused by various cytotoxic factors and plays a critical role in tumor cell apoptosis [25]. Interestingly, Bcl-2 also inhibits the transmembrane flow of Ca^2+^. Lam et al. have reported that the apoptosis induced by the calcium pump-specific inhibitor thapsigargin can be inhibited by Bcl-2. The reason is that Bcl-2 inhibits the transmembrane flow of Ca^2+^, suggesting that Bcl-2 can regulate apoptosis by regulating the intracellular Ca^2+^ [26]. Recent studies have shown that Bcl-2 family proteins interact with inositol 1,4,5-triphosphate receptor in endoplasmic reticulum (ER) to regulate its channel switch [27,28]. Anti-apoptotic proteins inhibit the excessive release of Ca^2+^ from ER and support cell survival, while pro-apoptotic proteins enhance the release of Ca^2+^ from ER and upregulate the concentration of Ca^2+^ in mitochondria and trigger apoptosis [27,29]. However, whether Bcl-2 affects CACNA1H activity and forms feedback regulation needs further experimental study.

Studies have shown that the inhibition of CACNA1H activates ERS, thereby inducing skeletal muscle atrophy [13]. ERS can promote the processing of unfolded or misfolded proteins in the ER, which facilitates the restoration of homeostasis and maintenance of cell survival, but persistent ERS leads to UPR and cell apoptosis [30]. The main ER transmembrane proteins involved in UPR signaling are PERK, IRE-1, and ATF6 [31]. Under normal conditions, they usually bind to GRP78 in an inactive state [32]. In the presence of ERS, GRP78 dissociates from these proteins and then binds to higher affinity unfolded and misfolded proteins. At the same time, PERK, IRE-1, and ATF6 dissociated from GRP78 were activated, eventually inducing UPR and apoptosis. Our results proved that the knockdown of CACNA1H and T-type Ca^2+^ channel inhibitor increased the expression of PERK, GRP78, CHOP, and ATF6, indicating the suppression of CACNA1H-activated ERS in U251 cells. Then, we found that ERS inhibitor UR906 blocked the apoptosis induced by CACNA1H inhibitor, suggesting that CACNA1H downregulation induced the apoptosis of tumor cells by activating ERS.

In addition, we also analyzed the effects of another T-type Ca^2+^ channel inhibitor, NNC55-0396, on the proliferation and ERS of glioma cells. The results confirmed that NNC55-0396 not only decreased cell activity, but also activated ERS in U251 cells. NNC55-0396 is a highly selective T-type calcium channel blocker for the Cav3.1 T-type channels, and its role in tumor cells is rarely reported. This result suggested that different subtypes of T-type Ca^2+^ channels might have a universal regulatory effect on ERS and apoptosis in glioma cells, which is worthy of our in-depth study. This result also indicated that NNC55-0396 might be considered as a new small molecule with anti-cancer effect.

## 5 Conclusion

Our data highlighted the effect of CACNA1H as an oncogenic gene in human glioma. Suppression of CACNA1H activated the ERS and thus induced apoptosis in glioma cells. T-type Ca^2+^ channel inhibitors ABT-639 and NNC55-0396 also induced apoptosis through ERS in glioma cells.
